# Protein Scaffolds Modulate the Electronic Structure of a Common Ketyl Radical Intermediate in Thiamine Pyrophosphate-Dependent Radical Biocatalysis: Insights from EPR and ENDOR Spectroscopy

**DOI:** 10.64898/2026.10.15.759499

**Published:** 2026-10-16

**Authors:** Guodong Rao, Zheng Zhang, Xin Liu, Angela A. Shiau, R. David Britt, Yang Yang

## Abstract

New-to-nature radical biocatalysis has emerged as a powerful strategy for asymmetric C–C bond formation, yet the enzyme-bound radical intermediates that underpin these transformations have remained largely uncharacterized. Here, we combine photobiocatalytic reaction development with multifrequency electron paramagnetic resonance (EPR), electron-nuclear double resonance (ENDOR) spectros-copy, and density functional theory (DFT) calculations to elucidate the ketyl radical intermediates formed during thiamine pyrophosphate (TPP)-dependent radical biocatalysis. We first develop an oxidative photobiocatalytic cross-coupling of aldehydes with alkyltrifluoroborates catalyzed by engineered benzoylformate decarboxylase (BFD) variants, providing enantioenriched α-chiral ketones through a new oxidative radical manifold inaccessible to benzaldehyde lyases (BALs). Freeze-quench multifrequency EPR and ENDOR spectroscopy directly identify the catalytically generated TPP-bound ketyl radical and reveal that this common radical intermediate adopts distinct electronic structures in BFD and BAL despite their identical chemical composition. Analysis of ^1^ H, ^19^ F, ^31^ P, and ^14^ N hyperfine interactions, together with DFT calculations, demonstrates that enzyme-specific active-site conformations redistribute spin density between the TPP cofactor and substrate fragment. Greater substrate-centered spin delocalization in BFD provides a mechanistic basis for its superior radical C–C coupling activity relative to BAL. These results establish a direct relationship between protein-controlled electronic structure and catalytic function in TPP-dependent radical biocatalysis and demonstrate that engineering the electronic properties of a common radical intermediate represents a useful strategy for controlling enzyme reactivity in radical biocatalysis.

**Table of Content:**

